# Elucidating the mechanism of traditional Chinese medicine formula (*Yifei-sanjie Pill*) in alleviating the chemobrain based on network pharmacology and experimental verification

**DOI:** 10.1016/j.jtcme.2024.11.008

**Published:** 2024-11-24

**Authors:** Yingchao Wu, Hui Wang, Zheng Liang, Jiaqi Cui, Siyan Liu, Yiliu Chen, Dajin Pi, Mingzi Ouyang

**Affiliations:** a School of Traditional Chinese Medicine, Jinan University, Guangzhou, Guangdong, 510632, China; b The Second Clinical Medical College of Guangzhou University of Chinese Medicine, Guangzhou, Guangdong, 510405, China; c State Key Laboratory of Traditional Chinese Medicine Syndrome, The Second Affiliated Hospital of Guangzhou University of Chinese Medicine, Guangzhou, Guangdong, 510120, China; d Department of Breast, Guangdong Provincial Hospital of Chinese Medicine, Guangzhou, Guangdong, 510120, China; e Guangdong Academy of Traditional Chinese Medicine, Guangzhou, Guangdong, 510120, China

**Keywords:** Traditional Chinese medicine formula, Chemobrain, Inflammation, Pyroptosis, Ferroptosis

## Abstract

**Background:**

Cisplatin (DDP) is widely used to treat clinical malignant tumors, but it is also accompanied by serious adverse reactions, such as brain nervous system injury. In clinical treatment and animal experiments, traditional Chinese medicine formula (*Yifei-sanjie Pill*, YFSJ) has been observed to relieve the symptoms of chemobrain caused by DDP chemotherapy, but its mechanism of action remains unclear.

**Methods:**

A subcutaneous model of non-small cell lung cancer was established in mice, which were subsequently treated with YFSJ to evaluate the mechanism by which YFSJ alleviates DDP-induced chemobrain. Specifically, the effects of DDP and YFSJ on the brain nervous system of mice were evaluated by behavioral tests, brain blood flow observation, network pharmacology analysis, and histological analysis. In addition, the damaging effect of DDP on neurons and the effect of YFSJ on neurons were verified by *in vitro* experiments.

**Results:**

YFSJ reduced systemic inflammation in tumor-bearing mice, restored the function of the blood‒brain barrier (BBB) to reduce the amount of cisplatin in the brain, alleviated the decreases in voluntary activity and brain blood flow microcirculation induced by DDP, and alleviated brain tissue pyroptosis and ferroptosis caused by DDP.

**Conclusions:**

YFSJ maintains the functional stability of the BBB by inhibiting tumor-induced inflammation, thereby preventing chemobrain induced by nervous system damage caused by DDP entering brain tissue.

## Graphical abstract

**Figure:**
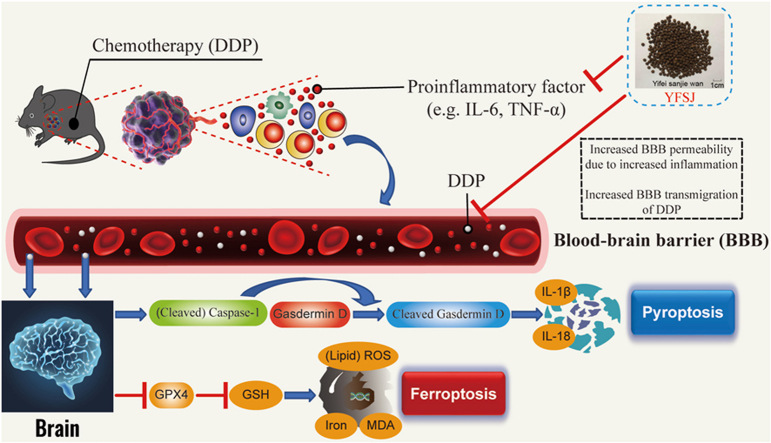

## 1 Introduction

Cisplatin (DDP) has played an important role in the clinical treatment of malignant tumors since it was first approved by the US Food and Drug Administration in 1978.^1^^,^^2^ According to the drug use statistics of drug manufacturers and hospitals, platinum-based chemotherapy drugs, represented by DDP, are used in 60–80 % of tumor chemotherapies in China.^3^^,^^4^ Although DDP is widely used clinically due to its good antitumor effect, numerous adverse reactions, such as renal toxicity,^5^ digestive toxicity,^6^ hematopoietic toxicity,^7^ ototoxicity,^8^ neurotoxicity,^9^ and allergic reactions,^10^ should not be ignored, as these conditions seriously limit the use of DDP and reduce patients' quality of life during treatment.

With the change in the concept of cancer treatment, cancer treatment involves not only the tumor itself but also the emotional changes experienced by patients during cancer.^11^^,^^12^ Emotional changes can be directly reflected in voluntary activity. Previous studies have shown that DDP treatment can reduce the voluntary activity of tumor-bearing mice but does not impair food-motivation.^13^ This phenomenon is one of the manifestations of chemobrain^14^, and although the specific mechanism of this phenomenon remains to be studied, we can assume that it may be closely related to nerve damage in the brain. In addition, another study has shown that neuroplasticity in the dentate gyrus (DG) of the hippocampus plays a role in regulating anxiety caused by adverse factors.^15^

Normally, DDP has difficulty crossing the blood‒brain barrier (BBB), so the amount of DDP entering brain tissue is much lower than that entering peripheral tissue.^16^ Studies have shown that when the body is exposed to high levels of inflammation, the permeability of the BBB changes,^17^ facilitating the ability of DDP to cross the barrier into brain tissue.^18^^,^^19^ After DDP enters brain tissue, due to its low clearance rate, it can cause damage to normal brain tissue cells,^20^ leading to normal cell apoptosis,^21^^,^^22^ pyroptosis^23^ or ferroptosis,^22^ etc., leading to a variety of adverse clinical symptoms.

Traditional Chinese medicine formulas (*Yifei-Sanjie Pill*, YFSJ) have been widely used in the clinic as adjunct drugs for the treatment of lung cancer and have achieved good curative effects, such as reducing tumor volume, relieving fatigue in patients, and prolonging survival time.^24^, ^25^, ^26^ Our previous study showed that YFSJ can reduce the serum levels of proinflammatory factors in tumor-bearing mice and thus reduce the systemic inflammatory load caused by tumors.^27^ In addition, we also observed in another study that YFSJ alleviated mental fatigue and resumed voluntary activity in tumor-bearing chemotherapy mice, but the specific mechanism has not been revealed.^28^ Therefore, further research on the mechanism of action of YFSJ in terms of clinical efficacy is helpful for clarifying the pharmacological mechanism of YFSJ to provide scientific evidence for the application of traditional Chinese medicine in clinical treatment.

Through *in vivo* and *in vitro* experiments, this study confirmed that YFSJ can reduce the serum inflammation level in tumor-bearing mice, thus maintaining the normal function of the BBB and preventing DDP from entering the brain tissue and causing chemobrain induced by damage to the brain nervous system.

## 2 Materials and methods

### 2.1 Animal ethics statement

A total of 40 female C57BL/6 mice weighing between 17 and 19 g and aged 6 weeks (Beijing HFK Bioscience Co., Ltd., Beijing, China) were used in this investigation. All the experiments were conducted according to the relevant laws and institutional guidelines. Mice were individually housed in independent ventilated cages at 24 °C–26 °C under constant humidity with a 12-h light/dark cycle. The mice were divided into 5 groups (n = 8), namely, the control, DDP, tumor, tumor plus DDP, and YFSJ groups, in a random order. The laboratory animal ethics committee of Jinan University approved the experimental procedure of this experiment(Approval No. IACUC-20200923-06).

### 2.2 YFSJ preparation

YFSJ (Cat. #Z20190015000) was purchased from the First Affiliated Hospital of Guangzhou University of Chinese Medicine (Guangdong, China). Each packet of YFSJ was 8 g, which was equivalent to 16.4 g of traditional Chinese medicine. The dosage of the subsequent treatments was the amount of herbal medicine. YFSJ was ground into powder using a Chinese medicine grinder and then dissolved in phosphate-buffered saline (PBS, Cat. #10010023; Gibco, NY, USA) at a final concentration of 1 g/ml (traditional Chinese medicine weight/volume).^28^ YFSJ was identified by liquid chromatography‒mass spectrometry (LC‒MS) according to previous methods, and the mass spectrometry results were analyzed via the mzCloud database (https://www.mzcloud.org/).

### 2.3 Chemicals, reagents and antibodies

DDP (Cat. #H20040813) was purchased from Jiangsu Hausen Pharmaceutical Co., Ltd. (Jiangsu, China). Primary antibodies against GFAP (Cat. #80788T), Iba1 (Cat. #17198T), Occludin (Cat. #91131T), Claudin-5 (Cat. #49564S), GPX4 (Cat. #59735S), and GAPDH (Cat. #5174T) and secondary antibodies anti-rabbit (Cat. #7074P2, 8889S and 4412S) were purchased from Cell Signaling Technology (Danvers, MA, USA). Primary antibodies against ZO-1 (Cat. #21773-1-AP), Caspase-1/p20/p10 (Cat. #22915-1-AP) and Gasdermin D (Cat. #20770-1-AP) were purchased from Proteintech Group, Inc. (Wuhan, China). Primary antibodies against VE-cadherin (Cat. #ab205336) were purchased from Abcam (Cambridge, UK).

### 2.4 Cell culture

Lewis lung cancer (LLC) cells (a non-small cell lung cancer cell line) were acquired from the Guangzhou University of Chinese Medicine (Guangzhou, China). HT22 cells (a mouse hippocampal neuron cell line) were acquired from Jinan University (Guangzhou, China). Dulbecco's modified Eagle's medium with high glucose, L-glutamine, and phenol red (DMEM, Cat. #11965092), fetal bovine serum (FBS, Cat. #10270106), penicillin/streptomycin (Cat. #10378016), and 0.25 % trypsin-EDTA with phenol red (Cat. #25200072) were purchased from Gibco (NY, USA). All cells were identified by short tandem repeat (STR) analysis. The cells were cultured in DMEM supplemented with 10 % FBS, 100 U/mL penicillin, and 100 μg/mL streptomycin. The cells were placed in a three-gas incubator (Thermo Fisher Scientific, Waltham, MA, USA) containing 5 % CO_2_ and 37 °C constant temperature and damp environment culture. The medium was changed every 72 h, and the cells were routinely subcultured when the cells reached 90 % confluence. Logarithmic growth phase LLC and HT22 cells were used for the experiments.

### 2.5 Animal experiments and sample collection

**Fig. 1:**
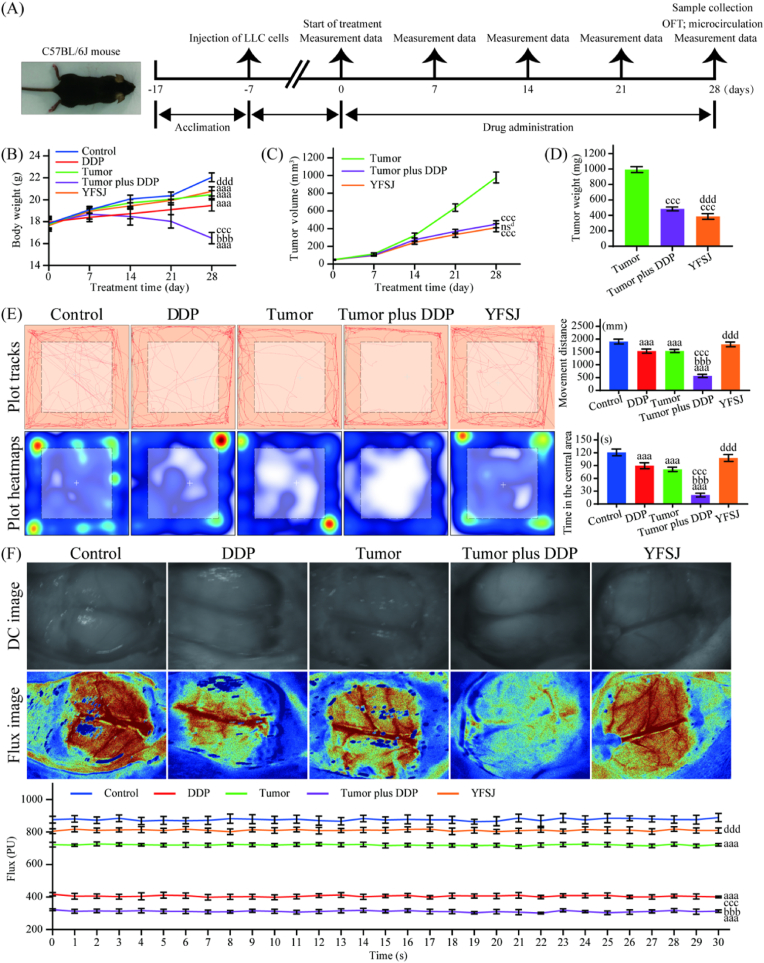
Effects of YFSJ on the growth, voluntary activity, and blood flow in the brains of chemotherapy mice. (A) The *in vivo* experimental protocol. (B) Body weight was measured every 7 days. (C) Tumor volume was measured every 7 days. (D) Xenograft tumor tissue weight after 28 days of treatment. (E) Plot tracks and plot heatmaps of the OFT as well as movement distance and central area residence time statistics. (F) Blood flow microcirculation in brain tissue and statistics of Flux values per second. The color represents the relative blood flow microcirculation, and the darker the red is, the greater the blood flow microcirculation. The data are presented as the means ± SDs; ^aaa^*P* < 0.001 compared with the control group; ^bbb^*P* < 0.001 compared with the DDP group; ^ccc^*P* < 0.001 compared with the tumor group; ^nsd^*P* > 0.05 and ^ddd^*P* < 0.001 compared with the tumor plus DDP group; n = 8.

The experimental protocol is shown in Fig. 1A. Mice in the tumor, tumor plus DDP, and YFSJ groups were given subcutaneous injections of 1 × 10^6^ LLC cells in the right flank to establish the xenogeneic mouse model of lung cancer. Mice in the control and DDP groups were injected with normal saline instead of LLC cells. Treatment was initiated seven days after LLC cell inoculation. Refer to previous studies to determine the optimal dose,^27^^,^^28^ mice in the DDP, tumor plus DDP, and YFSJ groups were intraperitoneal injected DDP (5 mg/kg, once weekly, total 5 times), mice in the YFSJ group was given YFSJ intragastric administration (4 g/kg, once daily, total 28 times), all treatments were controlled with normal saline instead of DDP or YFSJ. The body weights and tumor volumes were measured every 7 days, and the tumor size was calculated using the following equation: tumor volume = length × width^2^ × 1/2. The mice were sacrificed under anesthesia with pentobarbital (50 mg/kg, ip) after all *in vivo* experiments were completed, after which the plasma and brain tissue were collected for subsequent experiments.

### 2.6 Open field test (OFT)

The voluntary activity willingness and ability of the mice were measured by OFT after the completion of the last treatment according to the published protocol.^29^ In brief, the OFT was conducted in an arena made of plexiglass (100 × 100 × 50 cm^3^), and each mouse was placed in the center of the apparatus and analyzed for 5 min in a quiet room. The plot track and plot heatmap data were recorded and analyzed using EthoVision XT 14 software (Noldus Information Technology Co., Ltd., Beijing, China).

### 2.7 Real-time brain blood flow microcirculation imaging

The blood microcirculation in the brains of the mice was measured by a Moor full-field laser perfusion imager 2 (MoorFLPI-2; Moor Instruments, UK) after the completion of the last treatment according to the published protocol.^30^ In brief, after the mice were completely anesthetized, the head was stabilized on a mouse dissection table, the top of the head was disinfected, and a circular incision was made to fully expose the brain. Then, the brain was immediately scanned with a MoorFLPI-2, and the images were acquired in normal resolution mode at a rate of one frame per second. Finally, the manufacturer's software (MoorFLPI-2 Review, Version 5.0; Moor Instruments, UK) was used to analyze the images to assess brain blood flow microcirculation.

### 2.8 Histological analysis

The left hemispheres were removed from fresh brains, fixed in 4 % paraformaldehyde for 24 h, dehydrated, embedded in paraffin, and cut into 10 μm hippocampal coronal sections for subsequent experiments.

#### 2.8.1 Nissl staining

As previously reported,^14^ hippocampal coronal sections were stained with 0.5 % crystal violet (Cat. #C805211-25 g; Macklin, Shanghai, China) for Nissl staining. Changes in the morphology of neurons in the DG position of the hippocampus were observed under a light microscope at 200 × magnification.

#### 2.8.2 Fluoro-Jade B (FJB) staining

As previously reported,^31^ hippocampal coronal sections were stained with 0.0004 % FJB (Cat. #AG310-30 MG; Merck Millipore, Darmstadt, Germany) for FJB staining. Changes in the morphology of neurons in the DG region of the hippocampus were observed under a fluorescence microscope (Ex = 480 nm/Em = 525 nm) at 200 × magnification. All the sections were stained with DAPI to stain the nuclei.

#### 2.8.3 Luxol fast blue (LBF) myelin staining

The sections were rehydrated in xylene and descending grades of alcohol and subjected to LFB staining. LFB staining was performed according to the manufacturer's instructions (VitroView Luxol Fast Blue Stain kit, Cat. #VB-3006; Vitrovivo Biotech, Maryland, USA). Poststaining sections were observed to analyze the amount of myelin loss in the DG position of the hippocampus with the NIS Elements version 4.0 image analysis system (Nikon Instruments, Inc., Tokyo, Japan), Nikon Eclipse i5 (Tokyo, Japan) light microscope, and Nikon DS-Fi1c (Tokyo, Japan) camera attachment at 100 × magnification.

#### 2.8.4 Immunofluorescence

As previously reported,^32^ the sections were immunostained with the indicated primary antibodies, including anti-GFAP (1:100) and anti-Iba1 (1:100), followed by incubation with the corresponding secondary antibodies (1:500). Then, the sections were washed three times with TBST solution, after which the red or green fluorescence intensity was measured using a fluorescence microscope at 400 × magnification to determine the protein content of the sample. All sections were stained with DAPI for the nucleus in the tissues.

#### 2.8.5 Terminal-deoxynucleotidyl transferase-mediated nick end labeling (TUNEL) staining

After the sections were dewaxed, they were stained using a TUNEL apoptosis detection kit (Cat. #ATK00001; AtaGenix, Hubei, China) following the manufacturer's instructions. Then, the samples were stained with DAPI for 10 min and sealed after rinsing three times with PBS. Fluorescence images were captured at 400 × magnification under a fluorescence microscope (Nikon Eclipse ci, Japan).

#### 2.8.6 Transmission electron microscopy (TEM)

TEM sections of the hippocampus were prepared according to previously reported methods.^27^ The slices were observed and photographed under an electron microscope (HITACHI HT7700, Japan).

### 2.9 Network pharmacology analysis

#### 2.9.1 Screening for YFSJ-related active components and their targets

The Swiss ADME database (http://www.swissadme.ch/) was used to identify the active components of YFSJ, and 23 YFSJ-related active components were collected from this database (those with a drug likeness of YES and a bioavailability score ≥0.56). The Swiss Target Prediction database (http://www.swisstargetprediction.ch/) was subsequently used to identify potential targets of YFSJ. A total of 234 YFSJ-related targets were identified from this database (probability >0 %).

#### 2.9.2 Gene ontology (GO) and reactome pathway enrichment analysis

The online platform Xiantao Academic (https://www.xiantaozi.com) was used for pathway enrichment analysis of the targets of YFSJ, and the online platform NovoMagic (https://magic.novogene.com) was used to graph the enrichment analysis-related results.

#### 2.9.3 Protein‒protein interaction (PPI) network analysis and screening of core targets and hub genes of YFSJ

PPI network analysis of YFSJ targets was performed by using the String database (https://cn.string-db.org) with the organism “*Homo sapiens*” and a confidence score ≥0.4. Cytoscape software (version 3.9.0) was used to construct a “formula-component-target” network of YFSJ. To screen the core targets, the degree values of the targets of the PPI network were ranked using Cytoscape software, and the MCC algorithm in the cytoHubba plug-in of Cytoscape software was used to rank these targets.

### 2.10 Determination of inflammatory factor levels in serum and brain tissue

A mouse IL-6 ELISA kit (Cat. #EK206/3–96), mouse TNF-α ELISA kit (Cat. #EK282/4–96), mouse IL-10 ELISA kit (Cat. #EK210/4–96), mouse IL-1β ELISA kit (Cat. #EK201B/3–96), and mouse IL-18 ELISA kit (Cat. #EK218-96) were purchased from MultiSciences Biotech Co., Ltd. (Zhejiang, China). According to the manufacturer's instructions, the serum and brain tissue of the mice were analyzed via ELISA to determine the levels of inflammatory factors.

### 2.11 Determination of DDP content in brain tissue

As previously reported,^33^, ^34^, ^35^ the DDP concentration in brain tissue was determined via LC‒MS.

### 2.12 Detection of biochemical indices related to ferroptosis

A GSH and GSSG assay kit (Cat. #S0053; Beyotime Biotechnology, Shanghai, China) was used to measure GSH concentrations in brain tissue. An iron assay kit (Cat. #MAK025; Sigma, USA) was used to measure iron concentrations in brain tissue. A tissue ROS assay kit (Cat. #BB-470532; Bestbio, Shanghai, China) was used to measure ROS concentrations in brain tissue. A lipid peroxidation MDA assay kit (Cat. #S0131S; Beyotime Biotechnology) was used to measure the MDA concentration in the brain tissue. GSH, iron, ROS, and MDA levels in brain tissue were determined using these kits according to the manufacturer's instructions.

### 2.13 Protein content level analysis

As previously reported,^36^^,^^37^ the protein content of the sample was analyzed by western blotting (WB). Briefly, fresh brain tissue samples were lysed in RIPA buffer, the lysates were subsequently centrifuged at 12,000×*g* for 15 min at 4 °C, and the protein concentration in the obtained supernatant was determined using an enhanced BCA protein assay kit (Cat. #P0010S, Beyotime Biotechnology). Equal amounts of protein were separated via SDS‒PAGE and transferred to polyvinylidene fluoride (PVDF) membranes (Cat. #GVHP04700; Merck KGaA, Darmstadt, Germany). Next, the PVDF membranes were blocked with Tris-buffered saline plus Tween-20 (TBST) containing 5 % skim milk for 1 h and incubated with the indicated primary antibodies, including rabbit anti-ZO-1 (1:5000), anti-Occludin (1:1000), anti-Claudin-5 (1:1000), anti-VE cadherin (1:1500), anti-caspase-1 (1:2000), anti-Gasdermin D (1:2000), anti-GPX4 (1:1000), and anti-GAPDH (1:1000), after which the membranes were incubated with the corresponding secondary antibodies (1:5000) for 60 min at room temperature. The PVDF membranes were washed three times with TBST solution and visualized with a Beyotime's hypersensitive ECL kit (Cat. #P0018S; Beyotime Biotechnology). Finally, the density of the protein bands was determined via a gel image analysis system (ChemiDoxTM, Bio-Rad, USA). GAPDH was used as the loading control.

### 2.14 MTT colorimetric assay

Cell viability was measured by the MTT colorimetric assay. HT22 cells were seeded in 96-well plates (5 × 10^3^ cells/well in 100 μL) and cultured for 12 h. Then, the original medium was discarded, and 100 μL of DMEM-diluted DDP or YFSJ solution at different concentrations was added to each well. Alternatively, the cells were pretreated for 4 h with different inhibitors before the addition of DDP and/or YFSJ-containing medium. The cells were cultured with DDP and/or YFSJ-containing medium for 48 h before the MTT colorimetric assay was performed. After culture, 20 μL of MTT solution (5 mg/mL; Cat. #V13154; Gibco) was added to each well. After the cells were cultured for 4 h at 37 °C, the supernatant was discarded, 150 μL of DMSO was added to each well, and the sample was stirred well for 15 min. The absorbance of each well was then measured with a microplate reader (BioTek Epoch, Vermont, USA) at a wavelength of 490 nm. The recorded optical density (OD) values represent the cell vitality. The cell viability rate was subsequently calculated using the following equation: cell viability rate (%) = (OD_Sample_/OD_Control_) × 100 %.

### 2.15 Statistical analysis

All the data are presented as the means ± standard deviations and were analyzed with SPSS 13.0 software (SPSS, Inc., Chicago, IL, USA) and graphically visualized with GraphPad Prism 9 software (LLC, California, USA). Two-group comparison data were analyzed by one-way analysis of variance, and the repeated measures data were analyzed by repeated measures analysis of variance. Values of *P* < 0.05 were considered to indicate statistical significance.

## 3 Results

### 3.1 YFSJ alleviated voluntary activity inhibition and increased brain blood flow microcirculation induced by DDP chemotherapy in tumor-bearing mice

The *in vivo* experimental protocol is shown in Fig. 1A. As shown in Fig. 1B, both DDP (19.48 ± 0.51 g) and the tumor (20.47 ± 0.36 g) caused weight loss in the mice, and the weight loss was more pronounced when both were present (16.50 ± 0.52 g). Moreover, the experimental results showed that YFSJ (20.76 ± 0.42 g) can significantly reduce the weight loss of tumor-bearing chemotherapy mice. In addition, tumor volume and tumor weight were lower in the DDP (450.59 ± 40.50 mm^3^, 482.51 ± 24.73 mg) and DDP plus YFSJ (410.64 ± 46.39 mm^3^, 385.86 ± 36.49 mg) groups than in the tumor group (978.21 ± 62.18 mm^3^, 993.26.86 ± 37.78 mg) (Fig. 1C and D). The OFT results, which can reflect voluntary activity, showed that, compared with those in the control group (1904.28 ± 93.65 mm), the number of plot tracks reflecting movement distance was lower in the DDP (1531.43 ± 81.96 mm) and tumor (1535.39 ± 60.08 mm) groups but was significantly lower in the tumor plus DDP group (565.05.39 ± 62.65 mm). Similarly, the plot heatmaps that reflect the area of residence time showed that, compared with that of the control group (120.75 ± 7.94 s), the central residence time of the DDP (89.58 ± 6.97 s) and tumor (81.09 ± 5.09 s) groups was less reduced, while that of the tumor plus DDP group (21.01 ± 4.41 s) was significantly reduced. After YFSJ treatment (1793.36 ± 92.59 mm, 107.69 ± 8.08 s), these two behavioral indices were significantly improved in tumor-bearing chemotherapy mice (Fig. 1E). Observations of blood flow microcirculation in the brains of the study mice are shown in Fig. 1F. Data from the MoorFLPI-2 blood flow imager showed that the microcirculation on the surface of the brains of tumor-bearing mice treated with DDP (311.45 ± 11.90 PU) was significantly decreased compared with that in the control group (876.28 ± 23.55 PU). However, compared with that in the control group, the brain blood flow microcirculation in the DDP (405.29 ± 14.40 PU) and tumor (720.37 ± 11.53 PU) groups decreased, and compared with that in the tumor plus DDP group, the brain blood flow microcirculation in the DDP and tumor groups was still significantly greater than that in the tumor plus DDP group. Moreover, the brain blood flow microcirculation in the tumor group was significantly greater than that in the DDP group, and DDP obviously affected the blood flow of the microvessels, while simple tumors had little effect on the blood flow of the microvessels. After YFSJ treatment (810.78 ± 15.89 PU), the blood flow microcirculation in the brains of tumor-bearing DDP chemotherapy mice was obviously restored.

### 3.2 YFSJ reduced brain tissue damage caused by DDP chemotherapy in tumor-bearing mice

**Fig. 2:**
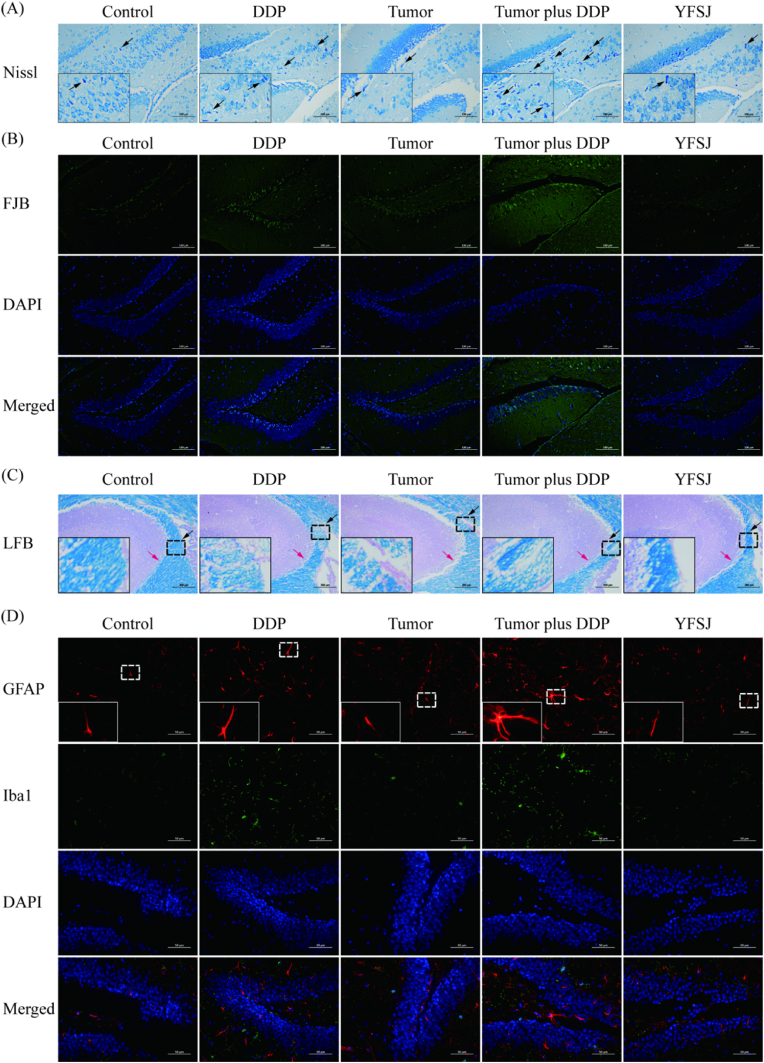
YFSJ reverses DDP-induced brain tissue damage. (A) Results of Nissl staining of the mouse hippocampus. The black arrow depicts the degenerative changes in the neurons in the DG sectors. Scale bar = 100 μm. (B) Effects of FJB staining on the hippocampus. Green fluorescence indicates degenerated neurons in the DG sectors. Scale bar = 100 μm. (C) Results of LFB staining of the hippocampus. The blank areas were stained to indicate myelin loss in neurons in the DG sectors. Scale bar = 200 μm. (D) GFAP and Iba1 immunostaining in the hippocampus. Merged overview of the relevant DG sectors with GFAP in red, Iba1 in green, and nuclear counterstaining with DAPI in blue. Scale bar = 50 μm.

**Fig. 3:**
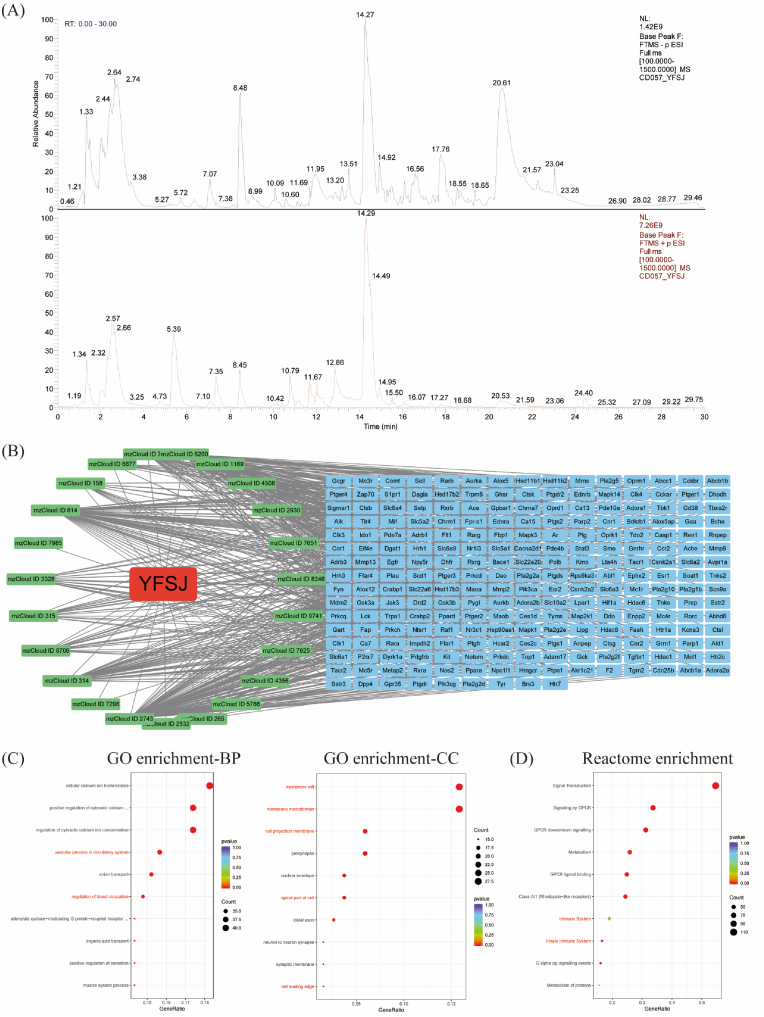
Harmacological targets of YFSJ and enrichment analysis of the targets. (A) Total ion current chromatogram of natural products identified by LC‒MS; column 1 (black) is the total ion current diagram in negative ion mode, and column 2 (red) is the total ion current diagram in positive ion mode. (B) Components of YFSJ and targets of their components. (C) The top 10 significantly enriched terms according to GO functional enrichment analysis are shown at the BP and CC levels, as was the Reactome pathway enrichment analysis.

Voluntary activity regulation is closely related to the structure and function of the DG positions in the hippocampus.^15^ First, Nissl staining of the hippocampus was performed in this study. As shown in Fig. 2A, in the tumor plus DDP group, there were severe degenerative changes in the neurons, as indicated by the morphology of the shrunken cytoplasm and condensed staining, compared with those in the control, DDP, and tumor groups in the DG positions of the hippocampus, whereas these signs of neural degeneration were alleviated in the YFSJ group. Fig. 2B shows representative images of pyramidal neurons from hippocampal DG positions stained with FJB. Many FJB-positive neurons were clearly observed in the tumor plus DDP group, and the number of FJB-positive neurons in the DDP group was slightly greater than that in the control group, while the number of FJB-positive neurons in the tumor group was not obvious. After YFSJ treatment, the number of FJB-positive neurons decreased significantly, indicating that the number of degenerated neurons decreased after treatment. The LFB staining results are shown in Fig. 2C. Compared with that in the control group, the area of the DG in the hippocampus in the DDP group, tumor group and tumor plus DDP group increased, indicating that the amount of myelin loss increased and that the most obvious change was in the tumor plus DDP group. Similarly, the loss of myelin in the DG region of the hippocampus was significantly reduced after YFSJ treatment. To investigate the extent of damage repair induced by damage to the central nervous system in mouse brains, GFAP was used to label activated astrocytes, and Iba1 was used to label microglia. As shown in the immunofluorescence image in Fig. 3D, GFAP immunoreactivity was increased in the DDP and tumor plus DDP groups in the DG region of the hippocampus, and it was more obvious in the tumor plus DDP group. The area with a positive expression of GFAP in the tumor group was not significantly greater than that in the control group. The staining results for Iba1 were similar to those for GFAP, and the number of Iba1-positive cells in the DDP-treated groups was significantly greater than that in the control group. Furthermore, the difference was more significant in the tumor plus DDP group, while the tumor alone did not have a marked effect on immunoreactivity in the brain. However, the YFSJ group had significantly fewer GFAP-positive and Iba1-positive cells than did the tumor plus DDP group.

### 3.3 Pharmacological targets of YFSJ and enrichment analysis of the targets

The LC‒MS primary ion current chromatogram of YFSJ is shown in Fig. 3A. Based on the prediction of the SwissADME database, a total of 23 compounds with medicinal potential in YFSJ were screened. In addition, 234 relevant targets of these active compounds were screened according to analysis of the Swiss Target Prediction database (Fig. 3B). The relevant YFSJ genes had 2659 associated terms at the BP level according to the GO enrichment analysis, 117 associated terms at the CC level according to the GO enrichment analysis, and 2250 associated terms according to the Reactome enrichment analysis. The top 10 significant terms at the BP level according to GO enrichment analysis, at the CC level according to GO enrichment analysis, and at the Reactome enrichment analysis are shown in Fig. 3C. The results of enrichment analysis showed that the BBB and inflammation-related pathways were significantly enriched.

### 3.4 Core target analysis of the components of YFSJ and the relationship between compound and target enrichment analysis

**Fig. 4:**
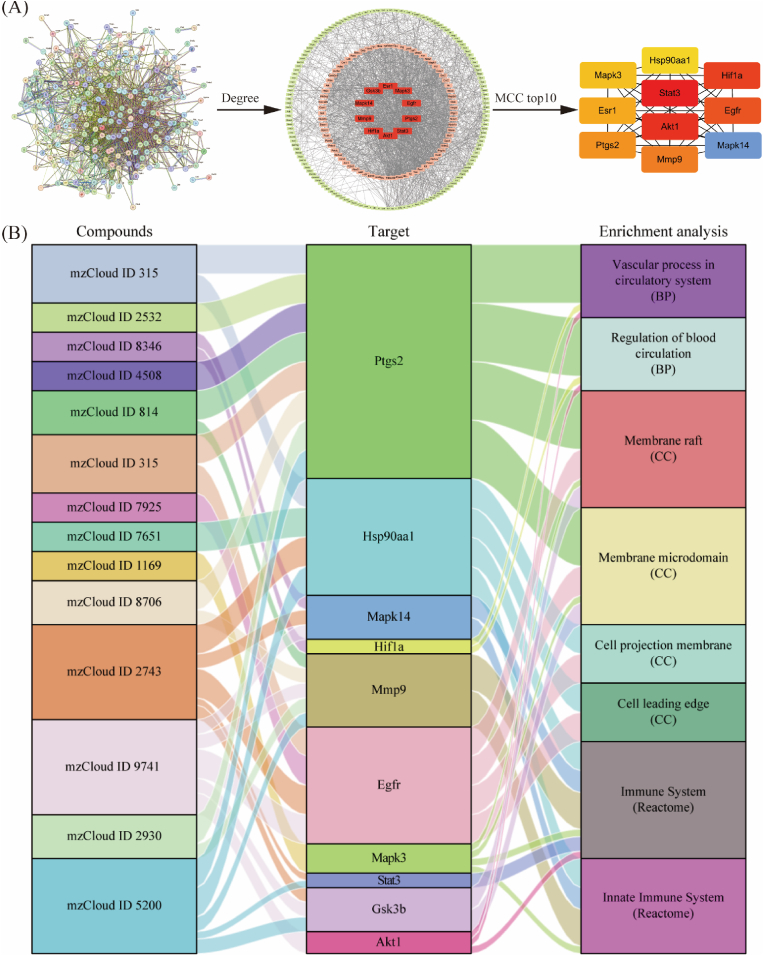
Core target analysis of the components of YFSJ and the relationship between compound and target enrichment analysis (A) PPI network analysis of YFSJ targets was performed by using the String database. The red nodes in the middle represent the larger degree values, and the small networks are the top 10 targets obtained by the MCC algorithm. (B) Sankey diagram of the compound-target-enrichment analysis results.

PPI network analysis was performed on these 136 targets using the String database, and the results are shown in Fig. 4A. As a result, 229 targets shared common nodes, the red nodes in the middle represented greater degree values, and the cyan nodes in the periphery represented smaller degree values. The MCC topology algorithm was subsequently used to determine the top 10 ranked nodes. Combining the degree values and the results of the MCC topology algorithm, Akt1, Stat3, Ptgs2, Egfr, Mapk3, Esr1, Gsk3b, Mapk14, Hif1a, Mmp9, and Hsp90aa1 were considered the core targets. The compounds and enrichment results related to the core targets were used to construct a Sankey diagram (Fig. 4B). The results showed that the active compounds participated in numerous enriched BBB and inflammation-related (8/9) pathways through the core targets (10/11).

### 3.5 YFSJ can reduce the levels of serum inflammatory factors, protect the BBB and reduce the concentration of DDP in brain tissue

**Fig. 5:**
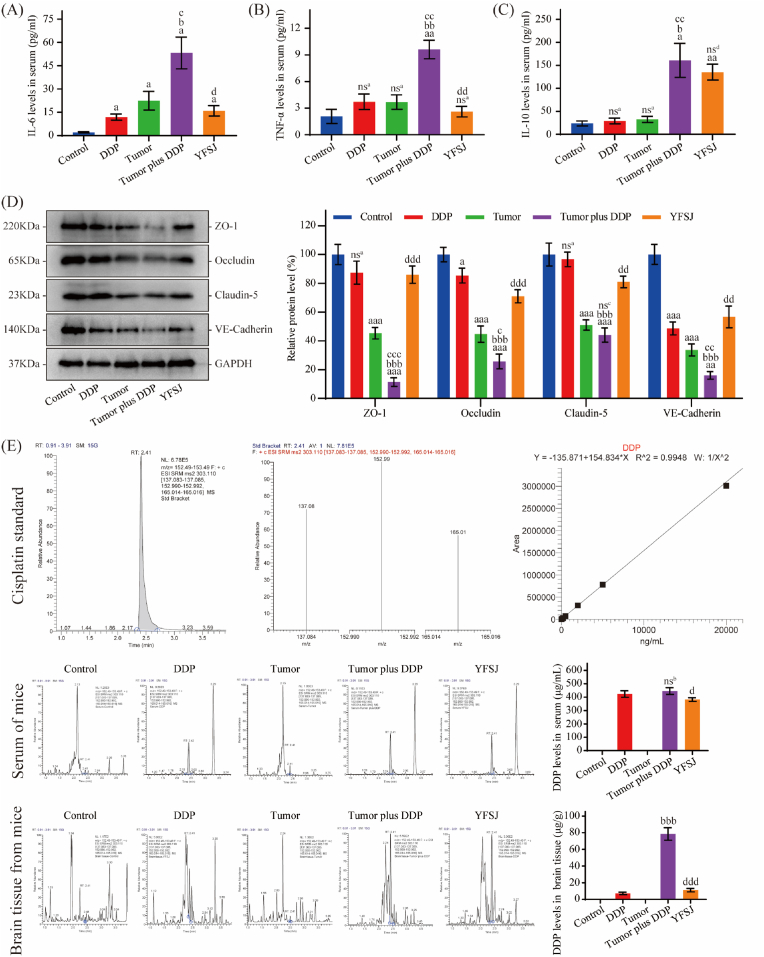
Effects of YFSJ on inflammation and the BBB. (A)–(C) The levels of inflammatory cytokines (IL-6, TNF-α and IL-10) in the serum were determined via ELISA. (D) Determination and relative quantification of BBB-related proteins. (E) The levels of DDP in serum and brain tissue were detected by HPLC. The data are presented as the means ± SDs; ^nsa^*P* > 0.05, ^a^*P* < 0.05, ^aa^*P* < 0.01, and ^aaa^*P* < 0.001 compared with the control group; ^b^*P* < 0.05, ^bb^*P* < 0.01, and ^bbb^*P* < 0.001 compared with the DDP group; ^nsc^*P* > 0.05, ^c^*P* < 0.05, ^cc^*P* < 0.01, and ^ccc^*P* < 0.001 compared with the tumor group; ^nsd^*P* > 0.05, ^d^*P* < 0.05, ^dd^*P* < 0.01, and ^ddd^*P* < 0.001 compared with the tumor plus DDP group; n = 3.

To determine the effects of tumor growth and/or chemotherapy on inflammatory cytokine levels, IL-6, TNF-α, and IL-10 levels in the serum were examined. The ELISA results for the serum samples are shown in Fig. 5A, B, and 5C. Compared with those in the control group (2.11 ± 0.41 pg/ml), the levels of the proinflammatory factor IL-6 were significantly greater in the DDP group (11.92 ± 2.01 pg/ml), tumor group (22.48 ± 6.04 pg/ml) and tumor plus DDP group (53.27 ± 10.21 pg/ml), and the levels in the tumor plus DDP group were significantly greater than those in the other two groups. In contrast to those in the IL-6 group, the proinflammatory factor TNF-α and the anti-inflammatory factor IL-10 were not significantly increased in the DDP (3.72 ± 0.87 pg/ml, 29.05 ± 6.16 pg/ml) or tumor groups (3.69 ± 0.81 pg/ml, 32.42 ± 6.58 pg/ml), but they were also increased in the tumor plus DDP group (9.61 ± 1.04 pg/ml, 160.94 ± 36.97 pg/ml). After supplementation with YFSJ (15.98 ± 3.36 pg/ml, 2.60 ± 0.59 pg/ml, 135.15 ± 17.19 pg/ml), the elevated levels of these proinflammatory factors were significantly reduced, but the levels of these anti-inflammatory factors were not significantly affected. We used WB to detect blood‒brain barrier-related proteins, and the results are shown in Fig. 5D. The expression of proteins that maintain BBB function was decreased in the tumor group and the tumor plus DDP group, and the above changes were reversed after YFSJ treatment. The DDP concentrations in the brain tissues of the different groups are shown in Fig. 5E. Compared with that in the DDP group (7.19 ± 1.49 μg/g), the content of DDP in the brain tissues of the tumor plus DDP group (78.51 ± 7.55 μg/g) was significantly greater, while the increase in DDP content was almost completely reversed after YFSJ treatment (11.14 ± 2.16 μg/g).

### 3.6 YFSJ reversed DDP-induced pyroptosis in brain tissue

**Fig. 6:**
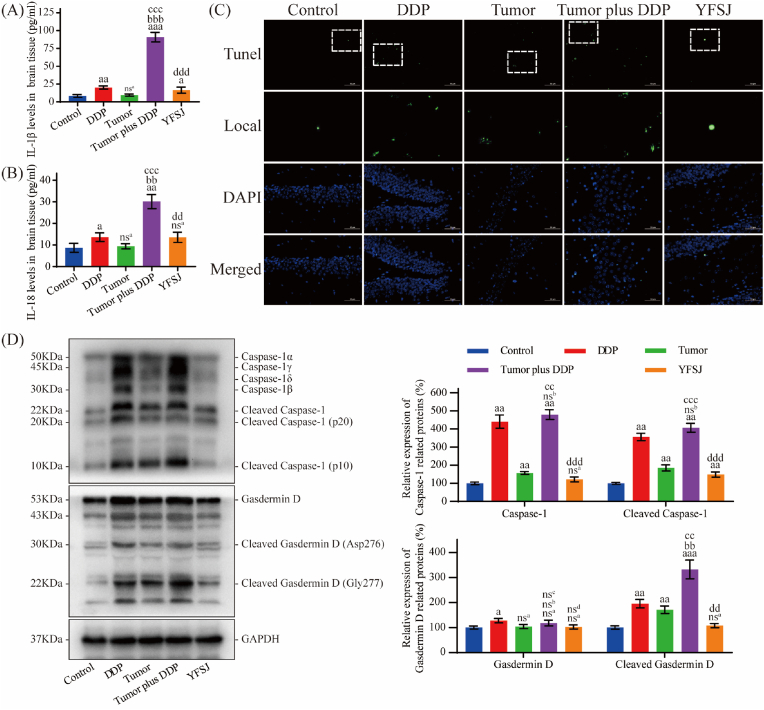
Effect of YFSJ on DDP-induced pyroptosis in brain tissue. (A)–(B) The levels of pyroptosis-related markers (IL-1β and IL-18) in brain tissue were determined via ELISA. (C) TUNEL staining of hippocampal neurons. Scale bar = 50 μm. (D) Determination and relative quantification of pyroptosis-related proteins. The data are presented as the means ± SDs; ^nsa^*P* > 0.05, ^a^*P* < 0.05, ^aa^*P* < 0.01, ^aaa^*P* < 0.001 compared with the control group; ^nsb^*P* > 0.05, ^bb^*P* < 0.01, ^bbb^*P* < 0.001 compared with the DDP group; ^nsc^*P* > 0.05, ^cc^*P* < 0.01, ^ccc^*P* < 0.001 compared with the tumor group; ^nsd^*P* > 0.05, ^dd^*P* < 0.01, ^ddd^*P* < 0.001 compared with the tumor plus DDP group; n = 3.

IL-1β and IL-18 are biochemical indicators of pyroptosis, and DNA damage is one of the characteristics of pyroptosis. Except TUNEL staining, which can be increased by both the tumor and DDP alone but is more severe by both agents, DDP or the tumor alone had little or no effect on these indicators, but the addition of DDP to tumors significantly affected the level of these biochemical indicators (*P* < 0.01) and pathological morphology. However, YFSJ treatment reversed the changes in these indicators (*P* < 0.01) and morphology caused by tumor plus DDP (Fig. 6A, B, and 6C). To further confirm the increase in pyroptosis in brain tissue in the tumor plus DDP group and the effective reversal of pyroptosis by YFSJ, proteins were extracted from brain tissues for analysis. WB analysis of protein of brain tissue indicated that pro-pyroptosis protein Caspase-1 (including cleaved Caspase-1) and cleaved Gasdermin D were slightly increased in tumor group, significantly increased in DDP group and tumor plus DDP group, and the latter increased more obviously, but there was no significant difference in the expression level of Gasdermin D protein among all groups, YFSJ treatment reversed these changes in the tumor plus DDP group (Fig. 6D).

### 3.7 YFSJ reversed DDP-induced ferroptosis in brain tissue

**Fig. 7:**
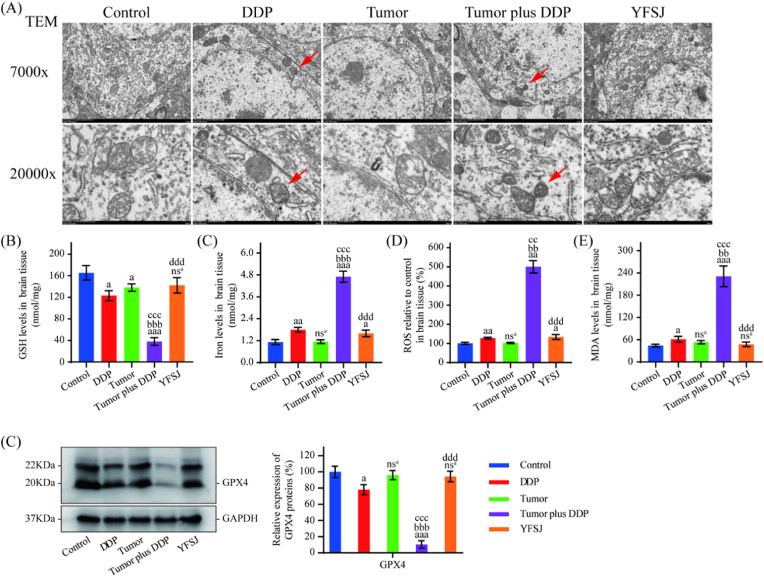
Effect of YFSJ on DDP-induced ferroptosis in brain tissue. (A) TEM images of hippocampal neurons. Scale bar = 2 μm or 500 nm. (B) (E) The levels of ferroptosis-related markers (GSH, iron, ROS and MDA) in brain tissue were determined via ELISA. (F) Determination and relative quantification of the ferroptosis-related protein GPX4. The data are presented as the means ± SDs; ^nsa^*P* > 0.05, ^a^*P* < 0.05, ^aa^*P* < 0.01, ^aaa^*P* < 0.001 compared with the control group; ^bb^*P* < 0.01, ^bbb^*P* < 0.001 compared with the DDP group; ^cc^*P* < 0.01, ^ccc^*P* < 0.001 compared with the tumor group; ^ddd^*P* < 0.001 compared with the tumor plus DDP group; n = 3.

GSH, iron, ROS and MDA are biochemical indicators of ferroptosis, and a decreased mitochondria density, increased membrane density, and reduced or absent mitochondrial ridges are characteristics of ferroptosis. Similarly, DDP or tumors alone had little or no effect on these indicators, but the addition of DDP to tumors significantly affected the levels of these biochemical indicators (*P* < 0.01) and pathological morphology. Fig. 7A, B, 7C, 7D, and 7E. Moreover, the trend in the expression of the antifunctional protein GPX4 was the opposite of that for the proptotic protein (Fig. 7C). However, YFSJ treatment was able to reverse the changes in the expression of these indicators (*P* < 0.001) and morphology caused by the tumor plus DDP.

### 3.8 YFSJ could not directly reverse DDP-induced neuron pyroptosis and ferroptosis *in vitro*

**Fig. 8:**
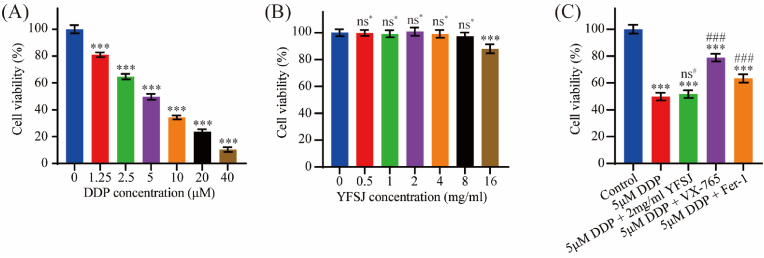
YFSJ could not directly reverse DDP-induced hippocampal neuron pyroptosis or ferroptosis *in vitro*. (A) HT22 hippocampal neuron reactivity to DDP. (B) HT22 hippocampal neuron reactivity to YFSJ. (C) Effect of YFSJ cotreatment and VX-765 or Fer-1 pretreatment on DDP-mediated inhibition of HT22 hippocampal neuron viability. The data are presented as the means ± SDs; ^ns^∗*P* > 0.05, ∗∗∗*P* < 0.001 compared with the control group (0 μM or mg/ml); ^ns#^*P* > 0.05, ^###^*P* < 0.001 compared with the 5 μM DDP group; n = 6.

*In vitro*, we verified the mechanism of action of DDP on hippocampal neurons and whether YFSJ directly acts on hippocampal neurons to reverse DDP-induced damage to hippocampal neurons. The results showed that DDP inhibited the cell viability of HT22 hippocampal neurons in a concentration-dependent manner (*P* < 0.001) (Fig. 8A), while YFSJ had almost no effect on the cell viability of HT22 hippocampal neurons at lower concentrations (Fig. 8B). Rescue experiments confirmed that the pyroptosis inhibitor VX-765 (*P* < 0.001) and the ferroptosis inhibitor Fer-1 (*P* < 0.001) could reverse the inhibitory effect of DDP on HT22 cells, while YFSJ (*P* > 0.05) could not directly reverse the inhibitory effect of DDP on HT22 cells (Fig. 8C). The results of *in vitro* experiments further confirmed that DDP can induce pyroptotic cell death and ferroptotic cell death in hippocampal neurons, while YFSJ does not directly affect hippocampal neurons to inhibit DDP-induced cell death.

## 4 Discussion

The use of DDP, a well-established and highly effective chemotherapeutic drug, especially for the treatment of lung cancer, tends to induce central nervous system damage and lead to mental symptoms such as lethargy and malaise.^38^, ^39^, ^40^ YFSJ is a traditional Chinese medicine commonly used in the adjuvant treatment of malignant tumors. It has good clinical therapeutic efficacy in treating tumors and improving quality of life.^24^, ^25^, ^26^ Although its antitumor efficacy has been well established, clinical observations have shown that YFSJ can effectively relieve adverse neurological symptoms such as mental fatigue in patients receiving chemotherapy.^41^ However, the mechanism of YFSJ in this regard has not been revealed, which is inconvenient for clinical use. Through network pharmacology and experimental verification, this study revealed that the inflammatory tumor microenvironment could promote the passage of DDP through the BBB and entry into brain tissue, leading to brain neuron damage. YFSJ can reduce the level of inflammation under tumor loading, stabilize the function of the BBB, prevent chemobrain caused by DDP entering brain tissue in large quantities, and ultimately protect brain tissue.

As a broad-spectrum antitumor drug, DDP can kill tumor cells efficiently but will inevitably cause damage to normal cells. Our experiments confirmed that DDP inhibited the viability of HT22 hippocampal neurons in a concentration-dependent manner. With the in-depth study of the mechanism of action of DDP, DDP has been found to kill cells, including normal cells, through a variety of pathways, including pyroptosis and ferroptosis.^42^ DDP can enter cells and directly act on GSH through GSH depletion,^43^ inhibit the activity of GPX4, produce many ROS and MDA molecules, and ultimately induce iron-dependent ferroptosis in cells.^32^ In addition, the large amount of ROS produced after DDP treatment increases the expression and activation of Caspase-1 to further cleave Caspase-1 into cleaved Caspase-1, which cleaves GSDMD to cleave GSDMD.^44^ Cleavage of GSDMD further promotes cell pyroptosis, and this process results in the production of large amounts of IL-1β and IL-18, ultimately leading to DNA damage.^44^ When neurons are damaged, astrocytes and microglias are activated, and damage is repaired.^45^ That is, the more severe the neuronal damage is, the greater the activation of astrocytes and microglia. Therefore, the percentage of GFAP- and Iba1-labeled microglia in the tumor plus DDP group was significantly greater than that in the control group. We confirmed that DDP-induced brain tissue damage was caused by both pyroptotic cell damage and ferroptotic cell damage and further confirmed that pyroptosis inhibitors and ferroptosis inhibitors could effectively reverse the inhibitory effect of DDP on neurons *in vitro*. Damage to the brain nervous system is usually accompanied by alterations in brain blood supply, especially capillary blood supply and voluntary activity,^46^^,^^47^ and the brain blood supply and the ability and willingness to volunteer activity of tumor-bearing mice are greatly reduced by DDP chemotherapy; these effects are fortunately largely restored by treatment with YFSJ. These findings indicate that YFSJ treatment effectively protected the brain tissue structure and the function of the brain nervous system in tumor-bearing chemotherapy mice.

Although the *in vitro* results showed that YFSJ could not directly reverse the inhibitory effect of DDP on neurons, our data showed that YFSJ could reduce the levels of proinflammatory factors produced by tumor burden without affecting the levels of anti-inflammatory factors, and our data also showed that YFSJ could greatly reduce the DDP content in the brain tissue of tumor-bearing mice. We believe that the reduction in the serum proinflammatory factor concentration may not be directly related to the reduction in tumor volume because although the tumor volume in the tumor plus DDP group was smaller than that in the tumor group, the level of inflammatory factors was greater, which may be related to the production of many proinflammatory factors during the process by which DDP kills tumor cells.^48^ Moreover, during inflammation, the levels of anti-inflammatory factors increase in response to heat treatment, which has been confirmed in previous studies.^49^ The decrease in the serum concentration of proinflammatory factors may be closely related to the active components of YFSJ. Network pharmacology analysis revealed that the core targets of the active ingredients of YFSJ were strongly correlated with inflammation and the BBB according to pharmacological enrichment analysis, suggesting that the reduction in serum proinflammatory factor levels and the stabilization of BBB function may be closely related to the active ingredients of YFSJ. We will further verify the effects of these components on the BBB in tumor-bearing chemotherapy mice in subsequent experiments. We will further verify the effects of these components on inflammation and the BBB and their relationship with tumor-bearing chemotherapy mice in subsequent experiments.

**Fig. 9:**
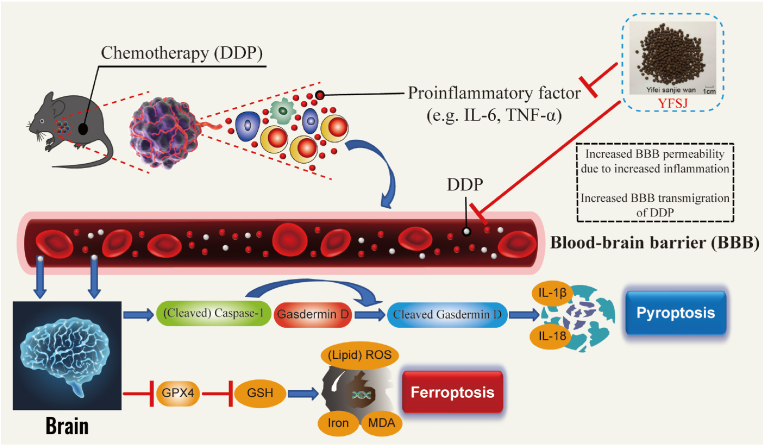
Schematic of the potential underlying mechanism by which YFSJ maintains the functional stability of the BBB by inhibiting tumor-induced inflammation, thereby preventing chemobrain induced by nervous system damage caused by DDP entering brain tissue.

Therefore, we can infer that during tumor chemotherapy, many proinflammatory factors are produced to destroy the integrity of the BBB, increase the permeability of the BBB to DDP, and lead to a large increase in the content of DDP in brain tissue, thereby inducing brain tissue damage. Damage to brain tissue affects the normal functions of the brain, such as the blood supply to the brain and voluntary activity regulation; thus, symptoms of chemotherapy in the brain appear. YFSJ can reduce the high level of inflammation caused by tumor chemotherapy, maintain the normal function of the BBB, reduce the concentration of DDP in brain tissue, and ultimately alleviate the damage to brain tissue during tumor chemotherapy. The hypothesized mechanism by which YFSJ reduces DDP-induced damage to the brain nervous system by reducing tumor-induced inflammation is shown in Fig. 9.

The results of this study can provide a basis for expanding the clinical indication of YFSJ. It is suggested that YFSJ may be suitable for the treatment of chemotherapy-related neurological side effects, and relevant clinical studies can be carried out in the later stage to validate the findings of this study. However, it is essential to acknowledge that some limitations persist. The precise molecular mechanisms underlying YFSJ's effects remain to be fully elucidated, and the long-term efficacy and safety in humans are still under investigation. Future research efforts should prioritize these areas to solidify the evidence for YFSJ's use in alleviating chemobrain and to provide a more comprehensive understanding of its mechanisms of action.

## 5 Conclusions

In this study, we innovatively found that the high level of inflammation induced by cancer chemotherapy could increase BBB permeability to chemotherapeutic agents. YFSJ maintains the functional stability of the BBB by inhibiting tumor-induced inflammation, thereby preventing nervous system damage caused by DDP from entering brain tissue.

## Availability of data

The datasets used and/or analyzed during the current study are available from the corresponding author upon reasonable request.

## Authors’ contributions

Yingchao Wu, Hui Wang, and Zheng Liang are co-first authors. Yingchao Wu and Hui Wang performed the *in vitro* experiments and wrote the manuscript. Jiaqi Cui performed the *in vivo* experiments. Zheng Liang was responsible for the identification and analysis of YFSJ. Siyan Liu completed the OFT and analyzed the data. Yiliu Chen completed the histological analysis. Dajin Pi and Yingchao Wu detected the biochemical indices. Yingchao Wu and Mingzi Ouyang designed the study and provided the initial idea. All the authors read and approved the final manuscript.

## Funding statement

This work was supported by the 10.13039/501100001809National Natural Science Foundation of China (Grant Nos. 81873155 and 81403340), which supported the design, analysis, and interpretation of the data in this study. The 10.13039/501100003785Medical Scientific Research Foundation of Guangdong Province of China (Grant NO. A2018006) and the Project of Administration of Traditional Chinese Medicine of Guangdong Province of China (Grant NO. 20191079) provided the animals, medicine, and other materials needed in the study.

## Declaration of competing interest

The authors declare that they have no known competing financial interests or personal relationships that could have appeared to influence the work reported in this paper.
